# Effect of experiential learning based AI‑generated aging video simulation on knowledge, attitude and gerontophobia in nursing students

**DOI:** 10.1186/s12912-025-04145-y

**Published:** 2025-12-12

**Authors:** Fatma Magdi Ibrahim, Ghada Shahrour, Suad Dukhaykh

**Affiliations:** 1 Faculty of Nursing, Mansoura University, Mansoura, Egypt; 2 RAK Medical and Health Science University, RAK College of Nursing, Rak, UAE; 3 Faculty of Nursing, Jordan University of Science and Technology, Irbin, Jordan; 4 RAK College of Nursing, RAK Medical and Health Science University, RAS Al-Khaimahh, UAE; 5 Management Department, College of Business, King Saud University, Riaydh, Saudi Arabia

**Keywords:** Aging, Experiential learning, AI simulation, Nursing students, Knowledge, Attitude, Gerontophobia

## Abstract

**Background:**

With global ageing accelerating, nursing students’ knowledge and attitudes toward older adults’ influence care quality. We evaluated an experiential learning–based, AI-generated ageing video simulation aligned with Kolb’s cycle.

**Design:**

One-group pretest–posttest quasi-experimental design with undergraduate nursing students at RAK College of Nursing (UAE). Gerontophobia paper Vancouver.

**Methods:**

Students completed baseline measures, viewed an AI-generated ageing simulation embedded in Kolb’s stages (concrete experience; reflective observation/abstract conceptualization; active experimentation), and repeated measures immediately post-intervention. Outcomes: knowledge (Palmore Facts on Aging Quiz), attitudes (Kogan’s Attitudes Toward Old People), and gerontophobia (Anxiety about Aging Scale). Paired tests assessed pre/post differences; effect sizes (Cohen’s d_av; Hedges’ g) summarized magnitude.

**Results:**

*N* = 107 (66.4% female, mostly 20–29 years). Knowledge increased 13.00 ± 3.00 → 22.18 ± 2.65 (*p* = 0.001; d = 3.25; g = 3.23). Attitudes improved 26.16 ± 6.38 → 33.17 ± 5.92 (*p* = 0.0002; d = 1.14; g = 1.13). Ageing-anxiety decreased 59.5 ± 16.2 → 50.3 ± 14.2 (*p* < 0.001; d = − 0.61; g = − 0.60). Directional sign indicates reduced anxiety.

**Conclusions:**

A brief, AI-generated simulation grounded in experiential learning substantially improved knowledge, attitudes, and reduced gerontophobia. Integrating such media into undergraduate curricula may strengthen gerontological competencies; longer follow-up and controlled comparisons are warranted.

## Background

Global demographic trends predict an exponential increase in the older adult population aged 60 and above in most countries worldwide by 2050 [1]. Aging can be a significant problem if society is not prepared to deal with its aging, presenting pejorative attitudes toward this phase of life [2]. It is expected to identify negative attitudes toward older adults in various sectors of society. These attitudes stem from ageism, which manifests in various forms, including stereotyping, prejudice, discrimination, marginalization, and disregard for older adults’ unique needs and contributions [3].

The World Health Organization’s analysis of the World Values Survey has revealed the widespread prevalence of negative or ageist attitudes toward older adults [4]. This comprehensive survey involved over 85,000 participants from 60 countries, uncovering significant variations in the roles assigned to older adults across different nations and cultures. These variations suggest that factors such as socioeconomic development, income inequality, the prominence of youth-oriented culture, and the breakdown of family support structures contribute to the levels of ageism observed in societies. Remarkably, the lowest levels of respect were reported in high-income countries [5].

Gerontophobia refers to an irrational fear, aversion, or discomfort towards aging and older adults. Individuals with gerontophobia may feel anxious about aging, dislike interacting with older adults, or avoid situations involving older people. This fear can be rooted in personal anxieties about the physical and cognitive decline associated with aging. Individuals who hold gerontophobic attitudes may be more likely to exhibit ageist behaviors, such as avoiding older adults or assuming they are frail or incapable. This contributes to a social environment that stigmatizes aging [6].

As of 2022, individuals aged 65 and older constituted approximately 1.4% of the United Arab Emirates (UAE) total population. Given the UAE’s estimated population of around 11 million in 2024, this percentage translates to approximately 154,000 older adults [7].

Older adults represent the leading group of users seeking healthcare and tend to have more extended hospital stays, significantly impacting healthcare systems’ overall financial costs [6]– [7]. Studies have shown that healthcare professionals’ negative attitudes toward aging lead to worse received care and poorer health outcomes for older adults [8]. Among the professions, nurses in the healthcare field are the best positioned to meet the growing demands imposed on the healthcare system by an aging society [9].

According to Cheng et al. (2024), nursing students’ attitudes toward older adults were considerably enhanced by simulation-based education. Additionally, the researchers revealed that when knowledge and attitude are considered, simulation in conjunction with conventional approaches works best [10]. Many conclusions and recommendations emerge from the literature on the use of AI in nursing education. Within his consideration of how AI could revolutionize nurse education, De Gagne [2023] emphasizes the importance of using AI sensitively and maintaining human interaction when providing personalized learning [11]. Božić (2024) proposed in an umbrella review that comprehensive approaches, including faculty development and curriculum design, are urgently needed to include AI into nursing education. Nursing schools are constantly challenged to develop curricula to prepare future nurses for managing, coordinating, and providing health care for older adults. Qualified nurses are needed to care for this complex population [12].

Nurses’ knowledge about aging, as well as nurses’ representations and attitudes toward older adults, are considered factors that affect the quality of health care. Studies on nursing students’ attitudes toward older adults have shown an association between negative attitudes and students’ lack of interest in pursuing nursing careers in gerontology [13]. In addition, there is evidence that interactions with older adults can effectively decrease ageism. Thus, it is essential to promote intergenerational contact [14].

The research illustrated that there is a lack of educational programs focusing on geriatric nursing care for all categories of nursing students who will deal with older people, and the healthcare given will be severely threatened by the negative stereotypic attitudes and misconceptions of nurses [15]. Developing a nursing workforce specialized in gerontological nursing will be challenging since there is already a shortage of skilled healthcare professionals globally. Failure to recruit and retain healthcare professionals in gerontological nursing is attributed to their negative attitudes toward aging and working with older adults. Fortunately. Education on aging was shown to decrease ageist attitudes and increase interest in working with older adults [16].

Enhancing knowledge about aging and fostering open, natural, and mutual relationships is an apparent necessity, which will ultimately reduce negative attitudes toward aging. Therefore, the study aims to evaluate the effectiveness of an AI‑generated aging video simulation, structured according to Experiential Learning Theory, in improving nursing students’ knowledge about aging, enhancing their attitudes toward older adults, and reducing gerontophobia.

## Research hypothesis

### H1 (Knowledge)

Participants’ scores on the Palmore Facts on Aging Quiz will significantly increase immediately after the Experiential Learning–based AI-generated aging simulation video intervention compared to baseline.

### H2 (Attitudes)

Participants’ scores on Kogan’s Attitudes Toward Old People Scale will be significantly better (more positive attitudes) post-intervention compared to pre-intervention.

### H3 (Gerontophobia)

Participants’ scores on the Anxiety Scale will significantly decrease (indicating reduced gerontophobia) immediately following the intervention compared to baseline.

### Theoretical framework

This study was based on Kolb’s Experiential Learning Theory, which offers a framework for comprehending the learning process [17]. We conceptualized this process as a four-stage cycle: concrete experience (CE), reflective observation (RO), abstract conceptualization (AC), and active experimentation (AE). While the RO stage concentrates on purposeful reflection after an encounter, the CE stage entails participating in an experience. AE is the process of putting those insights into practice by acting, while AC is the process of concluding the experience. Learning is a continuous cycle rather than a linear process, enabling students to revisit and improve on stages as they gain more knowledge and proficiency, which is a key component of Kolb’s theory. Kolb’s theory provided the pedagogical structure for sequencing simulation, guided reflection, conceptualization, and practice.

## Methods

### Design and participants

We conducted a one‑group pretest–posttest quasi‑experimental study at RAK Medical and Health Sciences University, RAK College of Nursing (January–April 2025) with Year 3–4 BSN students who had prior clinical exposure and no specialized training in gerontology.

### Target population

All eligible Year 3–4 BSN students enrolled in clinical rotations were invited ; 107 consented and completed both pre and immediate post assessments.

#### Inclusion

enrolled in Years 3–4; no specialized gerontology/elder‑care training; provided informed consent.

#### Exclusion

students on exchange programs or internships during data collection. A minimum required sample size of 107 was estimated.

#### Sampling and recruitment

Non‑probability convenience (classroom) sampling.

#### Determination of sample size

A priori sample size was estimated in GPower for a paired t‑test, two‑tailed, α = 0.05, power = 0.80, assuming a medium standardized mean difference (Cohen’s d = 0.50) informed by prior educational ageing interventions [18]. The final analytic sample (*N* = 107) exceeded this threshold.

#### Data collection tools

Data was collected using structured tools.

**Tool I: Demographic characteristics**: The questionnaire included demographic questions (age, gender, year of study).

**Tool II: Knowledge**: Palmore Facts on Aging Quiz (FAQ) [19, 20]. True/false items scored 1 (correct) or 0 (incorrect); totals reflect knowledge. Prior work reports acceptable internal consistency (α = 0.72). A scoring system was followed to assess the student’s knowledge of elderly care. Participants indicate whether each statement is true or false. Each correct answer is awarded one point, while incorrect answers receive zero points. The sum of correct responses represents the participant’s total score, reflecting their knowledge level about aging.

**Tool III: Attitudes: Kogan’s Attitudes Toward Old People (KAOP)** [21]. 34 items (17 positive; 17 negative), 6‑point Likert. Negative items are reversed; higher totals indicate more positive attitudes. Cronbach’s α = 0.764.

**Tool V: Gerontophobia: Anxiety about Aging Scale (AAS)** [22]; assesses anxiety and concerns about aging across physical, psychosocial, and loss domains; higher scores indicate greater anxiety (α = 0.85).

### AI intervention: technology and integration into kolb’s cycle

- **Authoring & rendering**: The video was generated using **text-to-video AI** with synthetic, non-identifiable characters depicting progressive ageing (sensory, musculoskeletal, respiratory, cognitive, social role changes). Scripts were evidence-informed and reviewed by gerontological nursing faculty for accuracy and tone. **No real patient data were used.**
- **Duration & delivery**: 15–20 min per session, delivered via the LMS/lecture theatre, over 3 weeks (2 sessions/week). **Closed captions** and pause/replay were enabled.

### Kolb alignment

#### Pre intervention

Prior to commencement of the intervention, a base line assessment of included participant’s knowledge, attitude and gerontophobia scores was carried out using Palmore Facts on Aging Quiz, Kogan’s Attitudes Toward Old People Scale and Aging Anxiety Scale.

**Intervention Phase** (implemented based on Kolb ELT): The intervention ran for 3 weeks, twice a week, approximately 30 min per session:

- Concrete Experience (CE): Participants watched the AI-produced aging simulation video for 15 to 20 min.
- Reflective Observation (RO) and Abstract Conceptualization (AC): After the video, participants completed a structured 5-minute guided debriefing and reflection, facilitated by the researcher.
- Active Experimentation (AE): Each session concluded with a brief 5‑minute case‑based application exercise focused on empathic communication and person‑centered care.

#### Post-Intervention (Follow-up Measurement)

Immediately after the intervention, the participants administering the same assessments used in the pre-assessment to gauge changes in knowledge, attitudes, and gerontophobia. Then, follow up two weeks after the intervention.

The detailed structure of the intervention, mapped to Kolb’s Experiential Learning Theory, is presented in Table 1.

**Table 1:** Study intervention. (adapted from kolb’s experiential learning theory)

Study Phase	Activity/Intervention	Theoretical Constructs	Measurement Tools	Data Collection Method
Pre-Intervention Assessment	Assessment of baseline knowledge, attitudes, and gerontophobia	AC, RO	- **Palmore Facts on Aging Quiz** (Knowledge)- **Kogan’s Attitudes Toward Old People Scale** (Attitudes)- **Aging Anxiety Scale** (Gerontophobia)	Online Survey
**Intervention Phase**				
Phase 1: Pre-Video Simulation Activities	Brief orientation to aging concepts (10–15 min lecture/readings)	AC, RO	N/A	N/A
	Initial knowledge reflection/discussion	AC, RO	Guided reflection prompts	In-person or online discussion
Phase 2: AI-Generated Aging Simulation Video Experience	Viewing the AI-generated aging simulation video (15–20 min)	CE	N/A	Video session (in-person or online synchronous)
	Structured debrief immediately after video	RO, AC	Guided reflection/debrief questions	Facilitated discussion (online survey or live discussion)
Phase 3: Active Experimentation (Post-Video Activity)	Planning empathic care scenarios or case study discussions	AE, AC, RO	Case scenarios & reflective prompts	Online or live workshop
Post-Intervention Assessment	Re-assessment of knowledge, attitudes, and gerontophobia	AC, RO	- **Palmore Facts on Aging Quiz** (Knowledge)- **Kogan’s Attitudes Toward Old People Scale** (Attitudes)- **Aging Anxiety Scale** (Gerontophobia)	Online Survey

### Statistical analysis

We used paired tests (α = 0.05) and report Cohen’s d_av for within-person change: d_av = (M_post − M_pre) /average (SD_pre, SD post), with Hedges’ g as the small-sample correction. For interpretability, we also provide percent change from baseline.

### Ethics approval and consent to participate

The study protocol was reviewed and approved by the RAK Medical and Health Sciences University (RAKMHSU) Health Ethics Committee, Ras Al Khaimah, United Arab Emirates (approval no. RAKMHSU‑HEC‑170‑UG‑N). All procedures were performed in accordance with the ethical standards of the institutional research committee and with the 1964 Declaration of Helsinki and its later amendments. Written informed consent was obtained from all participants.

## Results

### Demographic characteristics

A total of 107 nursing students participated in the study. Majority of participants were aged 20–29 years (91.6%), female (66.4%), and in their fourth academic year (66.4%). Most students (73.8%) reported no prior lectures or workshops related to aging (Table 2).

**Table 2:** Demographic characteristics of participants (*N* = 107)

Variable	Category	*n* (%)
**Age group**	< 20 years	7 (6.5)
	20–29 years	98 (91.6)
	30–39 years	1 (0.9)
	40 + years	1 (0.9)
**Gender**	Male	36 (33.6)
	Female	71 (66.4)
**Academic year**	3rd year	36 (33.6)
	4th year	71 (66.4)
**Prior ageing lectures/workshops**	**Yes**	28 (26.2)
	**No**	79 (73.8)

Notes: Values are counts and percentages. Percentages may not sum to 100 due to rounding

### Participants’ responses to knowledge items before and after the educational intervention

Pre-program responses revealed common misconceptions about aging. For instance, 70.1% of participants incorrectly believed that most individuals over 65 are senile, and 70.1% thought older adults have no interest in or capacity for sexual relationships. After the program, there was a substantial increase in the percentage of correct responses across nearly all items. For example, correct responses to the item regarding senility increased from 29.9% to 62.6%, and knowledge regarding older adults’ ability to engage in sexual relations improved from 29.9% to 65.4%. (Table 3)

**Table 3:** Distribution of responses to Palmore facts on aging items before and after the program

Knowledge statement	Pre: Correct *n* (%)	Pre: Incorrect *n* (%)	Post: Correct *n* (%)	Post: Incorrect *n* (%)
The majority of people over 65 are senile	32 (29.9)	75 (70.1)	67 (62.6)	40 (37.4)
All five senses tend to decline in old age	78 (72.9)	29 (27.1)	90 (84.1)	17 (15.9)
Most older adults have no interest in sexual relations	32 (29.9)	75 (70.1)	70 (65.4)	37 (34.6)
Lung capacity tends to decline in old age	74 (69.2)	33 (30.8)	87 (81.3)	20 (18.7)
Most older adults feel miserable most of the time	37 (34.6)	70 (65.4)	69 (64.5)	38 (35.5)
Physical strength tends to decline in old age	85 (79.4)	22 (20.6)	95 (88.8)	12 (11.2)
At least one‑tenth of the aged are in institutions	32 (29.9)	75 (70.1)	66 (61.7)	41 (38.3)

Abbreviations: n = number. The full item set is available upon request

### The overall mean knowledge score

**Fig. 1:**
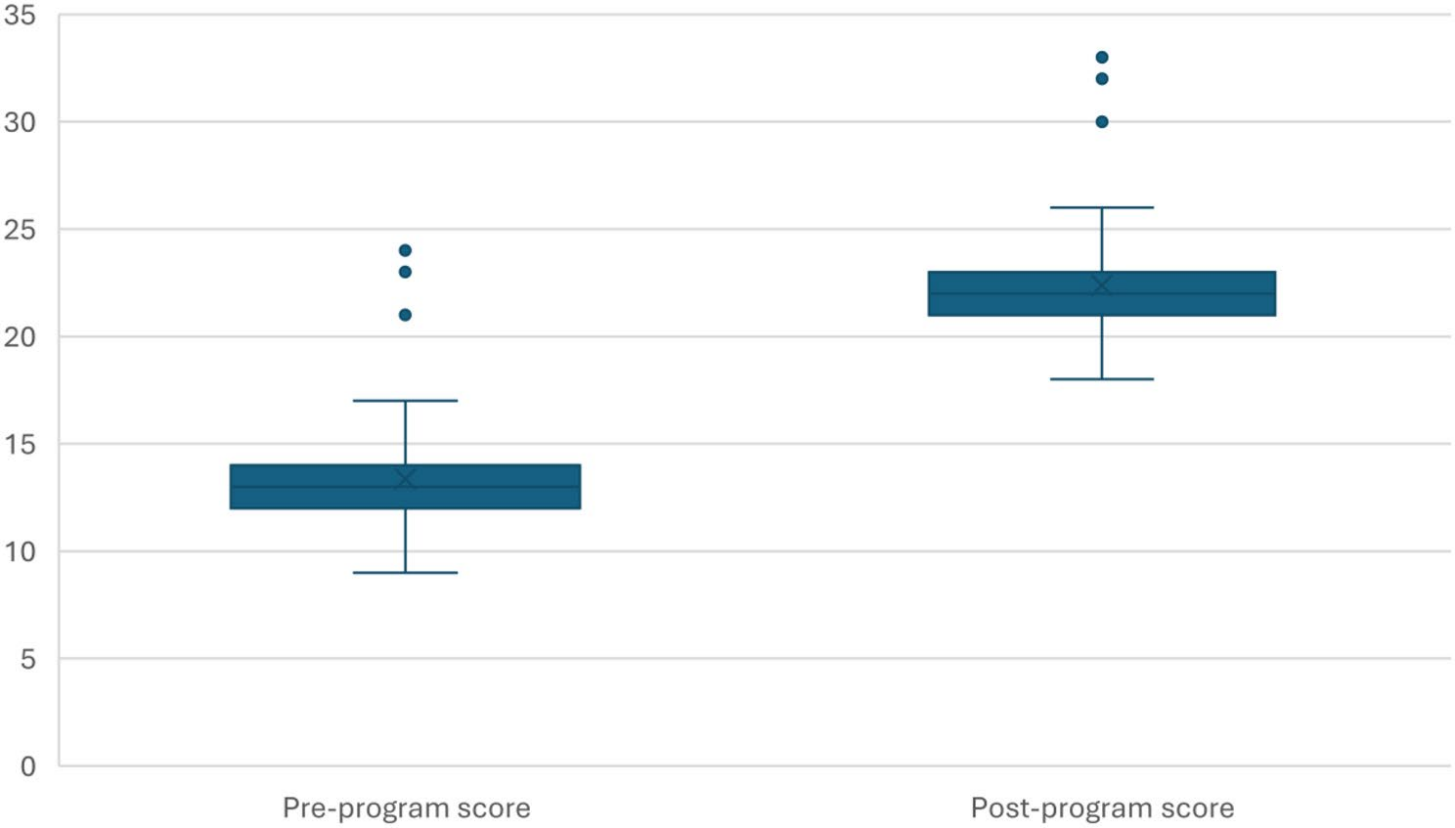
The sum knowledge score of participants (pre/post program comparison

Knowledge improved from 13.00 ± 3.00 to 22.18 ± 2.65 (*p* = 0.001; d_av = 3.25; g = 3.23; +70.6%), (*p* = 0.001), indicating a notable enhancement in students’ understanding of aging-related concepts (Fig. 1).

### Attitudes toward older adults

The educational program significantly improved students’ attitudes toward older adults. Positive attitude items such as “Most older adults are cheerful, agreeable, and good-natured” showed marked increases in mean scores (from 3.00 ± 1.00 to 3.61 ± 0.85; *p* = 0.008). Similarly, students increasingly disagreed with negative stereotypes. For instance, the item “Old people are usually lonely and unhappy” significantly decreased from 3.21 ± 0.97 to 2.40 ± 0.84 (*p* = 0.002) in this score. The overall attitude score increased significantly from 26.16 ± 6.38 to 33.17 ± 5.92 (*p* = 0.0002; d_av = 1.14; g = 1.13; +26.8%), (*p* = 0.0002), representing a more positive overall perception among older adults (Table 4).

**Table 4:** Selected kogan’s attitudes toward old people items: pre‑ and post‑intervention means (SD)

Item (examples)	Pre Mean ± SD	Post Mean ± SD	*p*‑value
Most older adults are cheerful, agreeable, and good‑natured	3.00 ± 1.00	3.61 ± 0.85	0.008
Old people are usually lonely and unhappy (rev.)	3.21 ± 0.97	2.40 ± 0.84	0.002
Most old people are difficult to get along with (rev.)	3.22 ± 0.94	2.39 ± 0.88	0.004
Total attitude score	26.16 ± 6.38	33.17 ± 5.92	0.0002

Notes: rev.=reverse‑scored. p‑values from paired t‑tests

### Anxiety about aging scale (AAS)

There was a significant overall reduction in fear of aging among participants, as reflected by the total fear of aging score, which decreased from 59.5 ± 16.2 to 50.3 ± 14.2 (d_av = − 0.61; g = − 0.60; −15.5%), (*p* < 0.001). All four domains of the Anxiety about Aging Scale (AAS) showed statistically significant improvements. In the “Fear of Old People” section, participants reported reduced discomfort and avoidance behaviors, as indicated by a decrease in total score from 14.18 ± 5.43 to 11.66 ± 4.80 (d_av = − 0.49), (*p* = 0.004). Psychological concerns such as fear of dependency, mental decline, and social isolation also declined significantly from 15.50 ± 4.34 to 12.17 ± 3.95, (d_av = − 0.80), (*p* = 0.001). Likewise, concerns related to physical appearance and frailty diminished from 14.64 ± 4.73 to 12.01 ± 4.15 (d_av = − 0.59), (*p* = 0.002), as did fears associated with loss of independence, relationships, and financial stability from 15.25 ± 4.58 to 11.98 ± 3.90 (d_av = − 0.77), (*p* = 0.003) (Table 5).

**Table 5:** Anxiety about aging scale subscales and total score: pre and post‑intervention means (SD)

Domain	Pre Mean ± SD	Post Mean ± SD	*p*‑value
Fear of Old People	14.18 ± 5.43	11.66 ± 4.80	0.004
Psychological Concerns	15.50 ± 4.34	12.17 ± 3.95	0.001
Physical Appearance Concerns	14.64 ± 4.73	12.01 ± 4.15	0.002
Fear of Loss	15.25 ± 4.58	11.98 ± 3.90	0.003
Total score	59.5 ± 16.2	50.3 ± 14.2	< 0.001

Notes: Lower post‑intervention scores indicate reduced gerontophobia. p‑values from paired t‑tests

## Discussion

This study showed significant and educationally meaningful improvements in ageing knowledge and attitudes, alongside reductions in ageing‑related anxiety, following a brief AI‑generated simulation grounded in experiential learning. Findings support integrating scalable, simulation‑based gerontological content into undergraduate nursing curricula. Future research should include controlled designs, longer follow‑up to assess durability, and exploration of mediators such as empathy and stereotype reduction. This finding is consistent with previous work that highlights technology-based interventions as practical approaches to promoting a deeper understanding of gerontological concepts among health professions students [23].

The findings are consistent with previous evidence that technology-supported learning games, such as simulations and visual narratives, can be effective mechanisms for increasing students’ knowledge about complex health topics related to ageing [24].

Studies by Dahlke et al. (2024) have noted that targeted educational programming has been shown to promote the development of gerontological knowledge among nursing students, particularly when targeted programs specifically focus on debunking myths and stereotypes associated with aging [25]. Our AI-generated video in the current study might have served the same purpose, being an attractive format for the facts.

### Knowledge gains

Our study also explored item-specific knowledge changes, revealing that participants’ understanding improved across a wide range of age-related topics, including misconceptions about cognitive decline, social engagement, physical health, and sexuality among older adults. These findings aligned with prior work identified widespread misconceptions among health professional students, particularly regarding the mental and emotional capacities of older adults [26]. For example, Pramanik (2022) found that students often believed aging to be uniformly associated with misconceptions of senility and social withdrawal, which were notably reduced following structured gerontological education [27]. Similarly, the present study’s correction of beliefs such as “most older adults are senile” or “most have no interest in sexual relations” reflects the power of educational media to challenge stereotypes and promote evidence-based thinking.

Our study further supports existing evidence that knowledge gains are robust in areas where pre-existing misconceptions are common. Myth items that targeted health and emotional well-being, as well as the ability to learn, work, and be productive in later life, witnessed the most significant improvements in scores. These results are consistent with those of White et al. (2024), who found that in nursing students, flexibility and continued learning in older adults are disregarded [28].

On the other hand, several knowledge items demonstrated minor improvements, indicating that some deep-seated beliefs, such as perceptions of religious behavior or institutionalization, may require a longer or experiential intervention to be changed entirely. This is supported by work from Burnes et al. (2019), who emphasized that while short-term educational programs can improve surface-level knowledge, addressing culturally ingrained stereotypes often demand longitudinal engagement and exposure to older adults in real-life settings [29]. However, the overall increase in all knowledge items obtained when comparing the first and last completions on most knowledge items used in our study demonstrates the positive bias due to the AI aging simulation.

### Attitudinal shifts

Our results suggest that incorporating AI-based educational media into the nursing curriculum may be a scalable and efficient strategy for enhancing gerontological competence. Masud et al. (2022) have advocated for more creative methods of teaching aging content, as lectures do not effectively address knowledge or attitudes [30]. The generalized gains in knowledge domains in our study lend support to the call to incorporate such techniques into formal healthcare education.

Additionally, improvements observed in students’ attitudes toward older adults following the intervention indicate a potential reduction in negative stereotypes or biases. This result is consistent with other studies [31], suggesting that even simulated contact, such as an AI-generated experiential video, can positively alter perceptions of stigmatized groups, thereby fostering more positive attitudes and empathy.

Our research revealed a significant positive shift in the attitudes of nursing students toward older adults after using an AI-generated educational intervention. The enhanced general attitude demonstrates that focused, technology-aided education can effectively impact learners\‘ stereotypes about aging, combating negative ones, and promoting empathic attitudes to others. This finding aligns with a previous study conducted by Martínez-Arnau et al. (2022), which found that structured gerontology curricula promote positive student attitudes, especially when interventions encourage active reflection and portray humanized images of older people [32].

### Reductions in gerontophobia

The item-specific improvements in our study further underscore that learners developed a greater appreciation for the sociability, emotional warmth, and cognitive potential of older adults. These results agree with those by Castro et al. (2023), who identified among nursing students’ negative and stereotypical beliefs about aging the expression that older persons are lonely, rigid, or experience a diminished cognitive function, until they were exposed to educational content that humanizes and dignifies aging [33]. In our intervention, we sought to address this misinformation by targeting the social skills and cognition of older adults.

Another result was a decrease in gerontophobia, as evidenced by the reduction in scores on the Aging Anxiety Scale following the intervention. Consistent with the current findings, previous research has also reported similar results, finding that experiential learning approaches to learning tend to significantly decrease anxiety and fear toward older adults, as these approaches foster empathy and perspective-taking (Kolb, 1984) [17].

Interestingly, the reduction in negative stereotypes was equally notable. The participants were increasingly dismissing the idea that older adults were a burden, complex, or unproductive. This finding is consistent with the results of Romaioli et al. (2021), who emphasized that ageist attitudes can only be modified through direct exposure to positive aging stories and by actively deconstructing society’s myths [34]. The AI-generated video in our study may have functioned as a powerful narrative tool, replacing deficit-based stereotypes with strengths-based perspectives of aging.

Although the findings are broadly consistent with the literature, some studies have shown mixed results. Fernández-Rodríguez et al. (2021) concluded that educational interventions led to improvements in knowledge, with a limited and sometimes contradictory impact on attitudes, unless there were real-life contact experiences with older individuals [35]. This implies that AI and digital storytelling (though potentially practical approaches) might be more effective when used in combination with experiential learning experiences, such as clinical rotations in geriatrics or intergenerational programs.

Our results supported the effectiveness of the educational intervention in reducing the fear of aging among nursing students, suggesting that AI intervention, in the form of educational games, could help alleviate gerontophobia. The decrease in overall fear, as well as in the specific domains of fear of older adults, psychological concerns, physical appearance concerns, and fear of loss, suggests that structured interventions can successfully reshape students’ perceptions and emotional responses to aging. This finding is consistent with the results reported by Diehl et al. (2020), who emphasized that education targeting misconceptions about aging can substantially reduce anxiety associated with the aging process [36].

The reduction in discomfort and avoidance behaviors toward older adults observed in our study supports earlier work by Bar-Tur (2021), who found that programs encouraging direct engagement and reflection on aging could mitigate negative emotional responses and foster greater comfort in interacting with older adults [37]. Furthermore, problems with aging-related issues, such as dependency and memory loss, are in line with evidence reported by Tinella and colleagues. (2023) posited that educational intervention could alleviate irrational concerns about becoming incompetent and losing autonomy among older adults [38].

The reduction in body-image concern is also consistent with those from Bashir et al. (2023), suggesting that negative body image associated with aging might be addressed through educational interventions that normalize physical changes and promote experience and wisdom over appearance [39]. Furthermore, problems with aging-related concerns such as dependency and memory loss are in line with evidence reported by Tinella and colleagues. (2023) posited that educational intervention could alleviate irrational concerns about becoming incompetent and losing autonomy among older adults [38].

The reduction in body-image concern is also consistent with that of Bashir et al. (2023), suggesting that negative body image associated with aging might be addressed through educational interventions that normalize physical changes and promote experience and wisdom over appearance [39].In our study, a significant decrease in fear of loss was also demonstrated, which included fears of losing independence, isolation, and economic stability. These findings are consistent with those of Jin et al. (2024), who suggested that this approach can help counter exaggerated fears of decline by promoting realistic and optimistic views of aging [40].

However, some studies, such as those by Bottomley et al. (2024), have noted that while knowledge about aging can improve, deep-seated fears and anxieties may persist unless reinforced with continuous exposure and positive aging experiences [41]. This suggests that while AI-driven educational programs are highly promising, integrating longitudinal experiences, such as service learning with older adults, might further consolidate the gains achieved.

## Conclusion & practical implications

An AI‑generated ageing simulation integrated within Kolb’s experiential cycle substantially increased knowledge, improved attitudes, and reduced ageing‑related anxiety among undergraduate nursing students. Given its brevity and scalability, programs can adopt the module as an adjunct to gerontological content to normalize ageing, counter pervasive myths, and rehearse empathic, person‑centered communication. Future controlled studies with longer follow‑up and intergenerational clinical components should assess retention and impact on clinical behaviors.

### Educational implications

Because the intervention is short, repeatable, and does not depend on access to high‑fidelity labs, it is feasible for integration across BSN curricula as an adjunct to lectures and clinical placements. Standardized debrief guides and case vignettes appear critical to translate “exposure” into conceptual change and skill planning.

### Limitations

One‑group pretest–posttest design without a control group limits causal inference; all outcomes were self‑report; the immediate post‑test timing emphasizes short‑term change; and the single‑institution, Year 3–4 sample may constrain generalizability. Although a two‑week follow‑up was planned, the present report focuses on immediate effects; longer‑term retention remains to be established.

### Future research

Randomized or quasi‑experimental comparisons (e.g., AI simulation vs. traditional lecture; AI + clinical exposure vs. clinical exposure alone), mixed‑methods evaluations to capture mechanisms (empathy, stereotype reduction), and intergenerational clinical experiences paired with the AI module are recommended to test durability and transfer to patient care.

## Data Availability

De‑identified data and analysis code are available from the corresponding author upon reasonable request and in line with institutional policies.
